# An Artificial Polyacrylonitrile Coating Layer Confining Zinc Dendrite Growth for Highly Reversible Aqueous Zinc‐Based Batteries

**DOI:** 10.1002/advs.202100309

**Published:** 2021-03-30

**Authors:** Peng Chen, Xinhai Yuan, Yingbin Xia, Yi Zhang, Lijun Fu, Lili Liu, Nengfei Yu, Qinghong Huang, Bin Wang, Xianwei Hu, Yuping Wu, Teunis van Ree

**Affiliations:** ^1^ Key Laboratory for Ecological Metallurgy of Multimetallic Minerals (Ministry of Education) School of Metallurgy Northeastern University Shenyang 110819 China; ^2^ China State Key Laboratory of Materials‐Oriented Chemical Engineering School of Energy Science and Engineering Nanjing Tech University Nanjing 210009 China; ^3^ National Energy Novel Materials Center Institute of Chemical Materials (ICM) China Academy of Engineering Physics (CAEP) Mianyang 621900 China; ^4^ Department of Chemistry University of Venda Thohoyandou 0950 South Africa

**Keywords:** dendrite suppression, polyacrylonitrile coating, zinc anodes, zinc‐ion batteries

## Abstract

Aqueous rechargeable zinc‐metal‐based batteries are an attractive alternative to lithium‐ion batteries for grid‐scale energy‐storage systems because of their high specific capacity, low cost, eco‐friendliness, and nonflammability. However, uncontrollable zinc dendrite growth limits the cycle life by piercing the separator, resulting in low zinc utilization in both alkaline and mild/neutral electrolytes. Herein, a polyacrylonitrile coating layer on a zinc anode produced by a simple drop coating approach to address the dendrite issue is reported. The coating layer not only improves the hydrophilicity of the zinc anode but also regulates zinc‐ion transport, consequently facilitating the uniform deposition of zinc ions to avoid dendrite formation. A symmetrical cell with the polymer‐coating‐layer‐modified Zn anode displays dendrite‐free plating/stripping with a long cycle lifespan (>1100 h), much better than that of the bare Zn anode. The modified zinc anode coupled with a Mn‐doped V_2_O_5_ cathode forms a stable rechargeable full battery. This method is a facile and feasible way to solve the zinc dendrite problem for rechargeable aqueous zinc‐metal batteries, providing a solid basis for application of aqueous rechargeable Zn batteries.

## Graphical abstract

A polyacrylonitrile (PAN)Z coating layer is employed to confine zinc dendrite growth for rechargeable aqueous zinc‐based batteries. The cyclability and Coulombic efficiency of the zinc anode porous PAN coating layer show that the polymer coating film is a feasible artificial membrane for a dendrite‐free zinc anode.

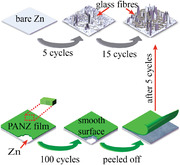

## 1 Introduction

The dwindling resources of fossil fuel and accelerating climate change are leading to urgent renewable energy demands. Harvesting sustainable energy from wind, sunlight, tides, and waves offers a great opportunity to overcome the energy shortage and catastrophic climate change. Collection and storage of the energy from these intermittent renewable resources is an intractable problem.^[^ ^1^ ^]^ Lithium‐based batteries (e.g., LIBs), which are among the most successful energy storage systems (EESs) in portable electronic devices such as laptops, smartphones, and wearable devices, have reached a bottleneck in aspect of large‐scale grids and electric vehicles due to the exhaustion of lithium resources (high cost) and the use of inherently flammable liquid organic electrolytes.^[^ ^2^ ^]^ Low cost, long life, and high safety are crucial factors for the successful application of the EESs.^[^ ^3^ ^]^ Aqueous batteries (ABs), as one of the most promising candidates for large‐scale EESs, have attracted the attention of researchers because of their low cost, environmental benignity, and safety.^[^ ^4^, ^5^, ^6^ ^]^ Because zinc metal has the highest theoretical specific capacity (820 Ah kg^−1^) among multivalent ABs and the abundant zinc supply (cost‐effective), aqueous rechargeable zinc‐ion batteries (ARZIBs) have been extensively investigated in recent years.^[^ ^7^, ^8^, ^9^, ^10^, ^11^, ^12^, ^13^ ^]^ However, dendrite growth on the zinc anode seriously impacts their reliability and limits their life‐span.^[^ ^14^ ^]^ Uneven zinc deposition during cycling is the main culprit, leading to the uncontrollable dendrite growth on the zinc anode's surface.^[^ ^15^, ^16^ ^]^ Therefore, homogenizing zinc deposition is the key technology to accelerate the large‐scale practical application of ARZIBs.

Considerable efforts in the realms of zinc electrode construction, electrolytes, and anode surface modification have been made to suppress dendrite growth. Porous 3D zinc foam is the classical electrode design for suppressing zinc dendrite.^[^ ^17^, ^18^, ^19^, ^20^ ^]^ Zn‐metal alloys were introduced as potential candidates for dendrite‐free zinc anodes.^[^ ^21^, ^22^ ^]^ Benefitting from rich deposition sites, multidimensional zinc hosts, such as 3D copper,^[^ ^23^, ^24^ ^]^ carbon nanotube (CNT) frameworks,^[^ ^25^ ^]^ carbon fiber mats,^[^ ^26^ ^]^ polyarylimide covalent organic frameworks (PI‐COFs),^[^ ^27^ ^]^ and porous carbonized metal–organic frameworks (MOFs),^[^ ^28^, ^29^ ^]^ have also been employed as current collectors to obtain dendrite‐free zinc anodes. Electrolyte engineering (electrolyte additives,^[^ ^30^, ^31^, ^32^, ^33^ ^]^ water‐starved electrolytes,^[^ ^34^, ^35^, ^36^, ^37^, ^38^ ^]^ deep eutectic solvent electrolytes,^[^ ^39^, ^40^ ^]^ (quasi‐) solid‐state electrolytes,^[^ ^41^, ^42^, ^43^, ^44^, ^45^ ^]^ alkaline‐mild hybrid electrolytes,^[^ ^46^, ^47^ ^]^ etc.) has also been shown to be an excellent way to introduce practical application of ARZIBs. Moreover, coating functional layers on zinc anode has been extensively developed to control the dendrite formation.^[^ ^48^ ^]^

Coating nanoscale electric materials on zinc surfaces is an effective method to evenly distribute charge, such as epitaxial graphene,^[^ ^49^ ^]^ metal nanoparticles,^[^ ^50^, ^51^, ^52^, ^53^ ^]^ and carbon/reduced graphene oxide (rGO)/CNT/graphite coating.^[^ ^37^, ^54^, ^55^, ^56^, ^57^ ^]^ Indeed, zinc deposition on these conductive coatings is more uniform and the dendrite growth is retarded to a certain degree, but dendrite formation might still be inevitable because of the uncontrollable electrochemical deposition. For this reason, some insulating materials, for example oxides (or sulfides)^[^ ^58^, ^59^, ^60^, ^61^, ^62^ ^]^ and MOFs^[^ ^63^ ^]^ have been coated on zinc surfaces to suppress dendrite formation by regulating the ion flux. Unfortunately, the brittle nature of these films restricts their application in large‐scale EESs.

To overcome the shortcomings of inorganic coating layers, researchers recently developed inorganic/MOF‐organic composites as coating layers to suppress dendrite growth, in which poly(vinylidene fluoride) (PVDF), the most popular organic binder, was used as coating preparation to strengthen the flexibility of blended films, such as PVDF–TiO_2_,^[^ ^16^, ^64^ ^]^ PVDF–CaCO_3_,^[^ ^65^ ^]^ PVDF–ZrO_2_,^[^ ^66^ ^]^ PVDF–kaolin,^[^ ^67^ ^]^ PVDF–MOFs,^[^ ^68^, ^69^ ^]^ etc. The inorganic/MOF–organic coating not only regulates ion transport, but also resists dendrite growth. Nonetheless, the high cost of nanoscale particles/ligands and the complicated preparation methods of MOFs will be obstacles to the commercialization of ARZIBs in large‐scale EESs. Moreover, the gap between binder and solid‐state particles probably makes the efforts to sift MOFs with precise Zn^2+^ transportation channels in vain.

Porous polymer coatings, a reasonable candidate to circumvent the abovementioned shortcomings, have rarely been reported because they are hydrophobic or water‐insoluble. Therefore, constructing hydrophobic polymer coating films with hydrophilic ion transit channels may be a useful solution to direct uniform deposition and control dendrite growth. Polyacrylonitrile (PAN), a widely used polymer, is used extensively as polymer matrix in LIB gel electrolytes, because of its chemical stability, excellent thermal stability, and high tensile strength and tensile modulus.^[^ ^70^, ^71^, ^72^ ^]^ By adapting to the volume change during metal plating/stripping, the PAN film's mechanical flexibility further confines uncontrollable zinc deposition.^[^ ^73^ ^]^ The polar nitrile group (‐CN), which is a linear structure, provides coordination sites to bridge Li^+^, Cu^2+^, and Zn^2+^ ions.^[^ ^74^, ^75^, ^76^ ^]^ However, the application of PAN in ARZIBs has not been investigated widely, probably because of the hydrophobicity of PAN membranes. Therefore, partly hydrophilic modification of the PAN coating layer would be a worthwhile way to address the dendrite issue on the surfaces of zinc anodes.

In this work, we investigate a polymer coating layer for a dendrite‐free zinc anode, which is prepared by dropping a PAN solution containing zinc trifluoromethanesulfonate (Zn(TfO)_2_) onto zinc foil (PANZ@Zn). Due to the addition of Zn(TfO)_2_, the PAN coating layer combined with a zinc salt (PANZ) has excellent hydrophilicity, which dramatically reduces the interfacial resistance of the Zn anode. With microchannels in the polymer network and the complexation effect between Zn^2+^ and the cyano groups (–CN), the PANZ coating layer does not only facilitate the uniform transport of dissolved Zn^2+^ in the PANZ membrane but also drives the uniform electrodeposition of zinc metal. A symmetric battery with a PANZ@Zn anode displays stable cyclability for more than 1140 h with a fixed capacity of 1 mAh cm^−2^. The full battery coupled with Mn‐doped V_2_O_5_ (MnVO) exhibits better electrochemical performance than that of the bare Zn anode, even when ultrathin zinc foil (20 µm) was employed in the full batteries.

## 2 Results and Discussion

**Figure 1:**
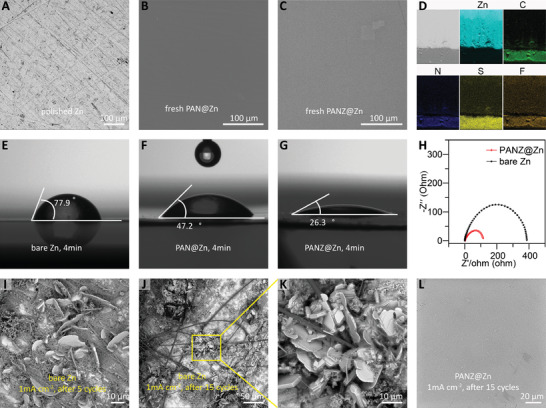
Morphologies, contact angles and EISs of different zinc anodes. A–C) Top‐view SEM images of bare Zn, PAN@Zn, and PANZ@Zn anodes. D) Cross‐section SEM image and EDS element mapping analysis of PANZ@Zn. E–G) Optical images of contact angles between different zinc anodes and electrolyte. H) Electrochemical impedance spectra (EIS) of symmetric cells with bare Zn and PANZ@Zn anodes. I) SEM image of flake‐shaped dendrites on bare Zn after 5 cycles at 1 mA cm^−2^ with 1 mAh cm^−2^. J,K) SEM image of dendrite clusters on bare Zn after 15 cycles at 1 mA cm^−2^ with 1 mAh cm^−2^. L) Top‐view SEM images of PANZ@Zn after 15 cycles at 1 mA cm^−2^ for 1 h.

**Figure** **1** shows the morphologies, hydrophilicity, and electrochemical impendence spectroscopy (EIS) of different zinc anodes. In order to remove the zinc oxide passivation layer, the bare zinc metal was polished with sandpaper before use (Figure 1A). As shown in Figure 1B, the surface of the zinc anode coating PAN layer without salt (aliased as PAN@Zn) looks smooth and dense, and is a little different from the surface of PANZ@Zn because of the microchannels formed in the film of PANZ@Zn (Figure 1C; and Figure S1, Supporting Information). The cross‐section scanning electron microscopy (SEM) images of bare Zn, PAN@Zn, and PANZ@Zn shown in Figure S1 (Supporting Information) show that the pure PAN coating layer is a pore‐free dense layer, and some micropores can be found in the PANZ layer containing zinc salt after the Zn(TfO)_2_ salt was partially removed by soaking in water for a few minutes. The energy‐dispersive X‐ray spectroscopy (EDS) of the Zn, C, N, S, and F elements confirms that the zinc salt in the PANZ coating layer is uniformly distributed (Figure 1D).

Since the uneven wetting of electrolyte on the zinc anode is the crucial factor that triggers the nonuniform distribution of charge and the nonhomogeneous electrodeposition on the solid‐electrolyte interface, the hydrophilicity of these zinc anodes in relation to electrolyte (2 m Zn(TfO)_2_) was investigated by measuring dynamic contact angles in the ambient environment. The initial contact angle of uncoated zinc was ≈87°, it was reduced to 77.9° in the following 4 min and then remained unchanged even after 20 min (Figure 1E; and Figure S2A, Supporting Information). In contrast, as illustrated in Figure 1F,G; and Figure S2B,C (Supporting Information), the contact angles of PAN@Zn and PANZ@Zn electrodes were reduced from ≈62.3° and ≈36.4° to 47.6° and 26.3° over 4 min, respectively. The distinct hydrophilicity improvement of PANZ@Zn is attributed to the microchannels formed by the introduction of Zn(TfO)_2_. It should be noted that the smaller contact angle of PAN@Zn than that of the bare Zn can be ascribed to the spreading of water on the PAN membrane.^[^ ^77^ ^]^ The PANZ membrane exhibits a high ionic conductivity (2.4 mS cm^−1^, Figure S2D, Supporting Information), which is similar to the previously reported,^[^ ^78^ ^]^ indicating the fast Zn^2+^ transport through the PANZ layer. The better wettability facilitates the even distribution of zinc‐ion flux on the zinc's surface during cycling, which reduces the ions’ motion resistance and promotes uniform zinc electrodeposition. The Nyquist curves of the impedance spectra of the different anodes before cycling reveal that the PANZ interfacial coating film significantly reduced the interfacial impedance of the zinc anode (Figure 1H). As shown in Figure S3A (Supporting Information), the highest resistance among the three anodes, displayed by PAN@Zn, substantially confirms that the pure PAN membrane without any additives is pore‐free and ion‐blocking in aqueous systems.

Bare zinc suffers from dendrite formation during cycling, usually after just a few cycles. In a study of the morphologies of the different zinc anodes before and after cycling, an in‐depth analysis shows how the PANZ coating layer tackles the dendrite problem. After five cycles at a current density of 1 mA cm^−2^ with a capacity of 1 mAh cm^−2^, many dendrites were found on the surface of the Zn anode (Figure 1I), and the dendrites had grown considerably and gradually gathered to form dendrite clusters after 15 cycles (Figure 1J). Many glass fibers embedded in the dendrites indicate that the dendrites penetrated the glass fiber membrane (Figure 1J,K).

The cycling performance of a symmetrical cell with the PAN@Zn anode is shown in Figure S3B (Supporting Information). The battery failed after working only 17 h because of an internal short circuit, with a bigger polarization than that of the bare Zn and PANZ@Zn. The zinc anode and separator of the failed PAN@Zn cell was disassembled for further investigation. Dendrites are evident on the edge of the zinc disk, which pierced into the glass fiber membrane and triggered the internal short circuit (Figure S3C–K, Supporting Information). In contrast, the PANZ@Zn anode showed a smooth surface without dendrite formation after 15 cycles (Figure 1L), showing that the addition of Zn(TfO)_2_) not only improves the hydrophilicity of a zinc anode with PAN coating layer but also improves the conductivity of Zn^2+^ in a PAN membrane, which boosts the uniform deposition of Zn^2+^ on the zinc electrode surface, consequently inhibiting dendrite formation.

**Figure 2:**
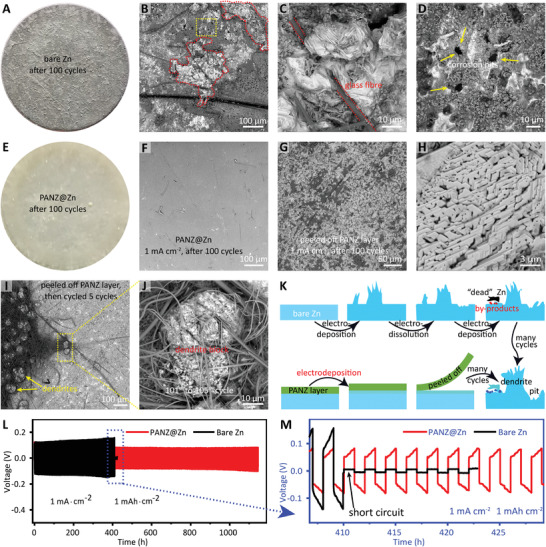
Morphologies of Zn anodes disassembled from PANZ@Zn||bare Zn symmetric cell stopped after the 100th cycle, and long‐term cycling stability of symmetric cells. A) Digital picture of bare Zn anode side. B–D) SEM images of bare Zn anode; bump‐like zinc dendrite in the red dashed‐dotted region; corrosion pits in the bright yellow square. C) Magnification of SEM image of glass fiber embedded into the bump‐like zinc dendrite. D) Erosion pits on the bare Zn anode. E) Digital picture of PANZ@Zn side. F) Top‐view SEM image of PANZ@Zn. G) SEM image of PANZ@Zn anode peeled off the PANZ coating layer. H) Magnified SEM image of PANZ@Zn anode peeled off PAN the coating layer. I,J) SEM images of bulk‐shaped dendrites on PANZ@Zn‐2 anode. K) Schematic illustration of dendrite formation process on bare Zn and PANZ@Zn‐2 electrodes. L,M) Voltage–time curves of bare Zn||bare Zn and PANZ@Zn||PANZ@Zn symmetric cells at 1 mA cm^−2^ with a fixed capacity of 1 mAh cm^−2^.

To further accurately study the dendrite suppression effect of the PANZ coating layer on the Zn anode, we tested a symmetrical cell consisting of bare Zn and PANZ@Zn electrodes at the current density of 1 mA cm^−2^ with a capacity of 1 mAh cm^−2^ for 100 cycles. Aside from the synergistic effect between the two electrodes, we also investigated the morphology change and the electrochemical performance of these two electrodes, which suffered almost identical plating/stripping electrochemical processes (**Figure** **2**). Many glass fibers can be seen adhering to the surface of cycled bare zinc because of the formation of dendrites piercing the separator (Figure 2A). Figure 2B–D shows the spike‐shaped dendrites and many corrosion pits on the bare Zn anode surface, suggesting that the electrodeposition and stripping processes occur at preferred prereaction positions to minimize the free energy of the microsystem.^[^ ^15^, ^21^, ^78^ ^]^ In contrast, the surface of PANZ@Zn remained flat, and the PANZ film stayed intact and tightly adhering to the zinc surface (Figure 2E,F).

The PANZ coating layer on PANZ@Zn was peeled off to reveal the morphology change of the zinc metal, and the SEM image shows a smooth surface without dendrite (Figure 2G). The magnified SEM image in Figure 2H shows that zinc was deposited orderly on the surface, indicating that the deposition behavior of Zn on the PANZ@Zn anode is different from that on its bare counterpart. The coating layer on the cycled PANZ@Zn anode was peeled off and then the “bare zinc” (denoted as PANZ@Zn‐2) was reassembled into a coin cell with fresh bare Zn and continuously cycled for another five cycles to further verify the function of the PANZ coating layer. The digital picture displays glass fiber sticking to the surface of the PANZ@Zn‐2 anode (Figure S4, Supporting Information), and in the magnified SEM images (Figure 2I,J) many protruding dendrites can be seen on the surface. We also evaluated the anticorrosion capability of the PANZ‐coated zinc anode. The Tafel curves measured in 2 m Zn(TfO)_2_ at a scan rate of 2 mV s^−1^ show the more negative corrosion potential of PANZ@Zn (Figure S5, Supporting Information), which implies that the PANZ coating layer can alleviate the hydrogen evolution reaction.

These results confirm that the PANZ coating layer can improve the surface wettability of the zinc electrode, reduce the interfacial impedance, relieve the corrosion of zinc, and confine the zinc ion flux to inhibit the growth of zinc dendrite.^[^ ^79^ ^]^ Based on the above results, we can depict schematically the zinc stripping/plating behavior on the surface of bare Zn and PANZ@Zn (Figure 2K). The poor cycling performance of the bare zinc electrode is mainly due to its severe interfacial reaction with water or dissolved O_2_ and the random deposition/stripping positions and the accumulation effect of zinc dendrite.^[^ ^80^ ^]^ In contrast, benefiting from excellent flexibility and hydrophilicity, the PANZ coating layer can not only adapt to the volume changes during the processes of zinc deposition/stripping and contain dendrite formation, but also facilitates uniform electrodeposition.

To evaluate the plating/stripping stability of the bare Zn and PANZ@Zn anodes, we investigated the long‐term galvanostatic cycling performance of symmetrical cells at a current density of 1 mA cm^−2^ with fixed capacity of 1 mAh cm^−2^. As illustrated in Figure 2L,M, the voltage hysteresis of the Zn||Zn cell exceeds 100 mV, and internal short‐circuit signals (sudden voltage drops) were already observed after about 410 h. In contrast, the PANZ@Zn||PANZ@Zn cell has a stable voltage profile for 1145 h with only 75 mV voltage hysteresis. The performance of PANZ@Zn anode is superior to the most previous works. As summarized in Table S1 (Supporting Information), some of previous zinc anodes presented excellent cycling stability under high current density, but the capacity of per cycle they delivered is too low to satisfy the practical application.^[^ ^81^, ^82^ ^]^ On the other hand, although some of anodes worked at higher current density, they could only deliver limited number of cycles.^[^ ^83^ ^]^

**Figure 3:**
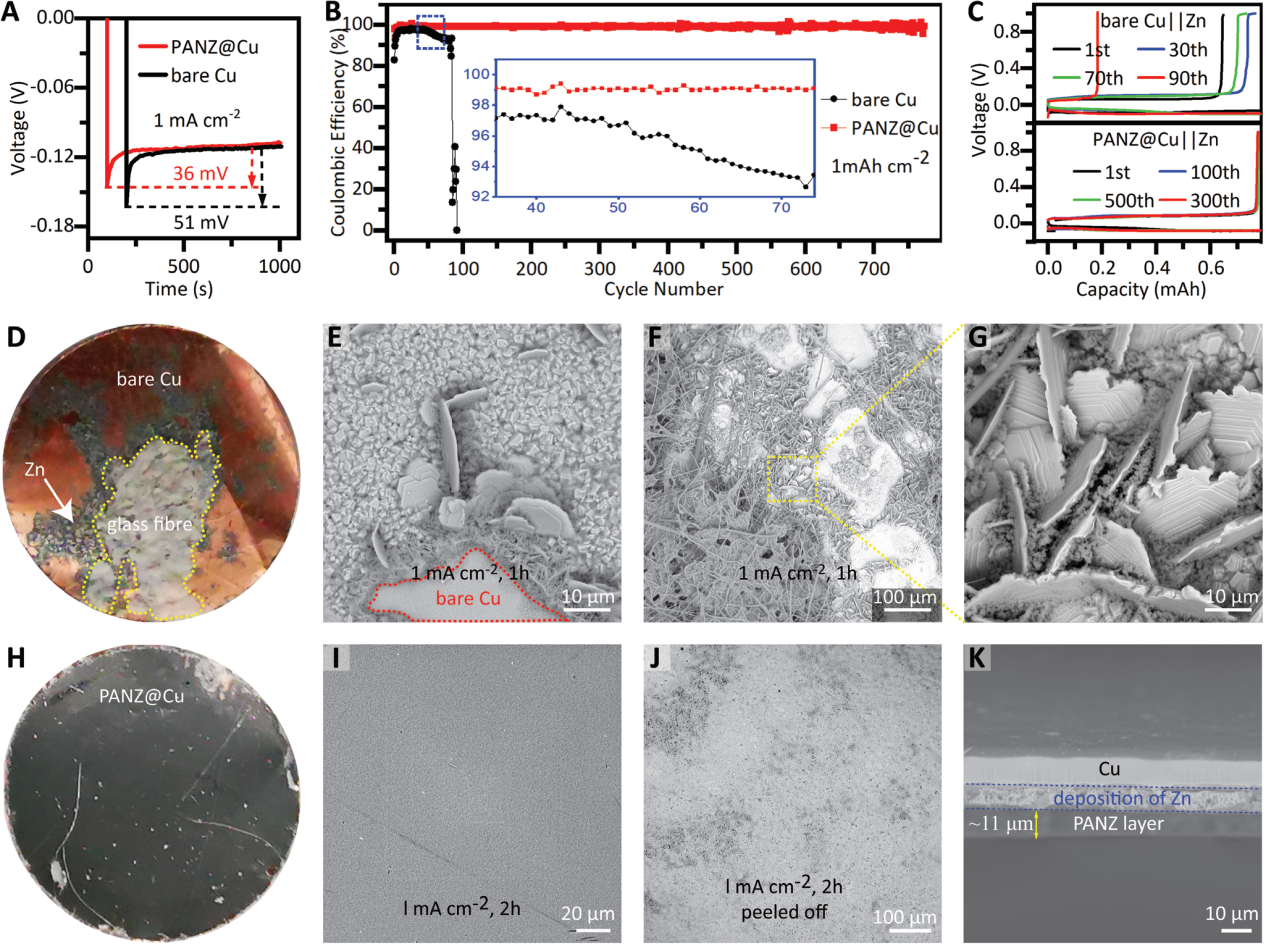
Overpotentials, Coulombic efficiencies, voltage–time curves and morphology analysis of PANZ@Cu and bare Cu electrodes. A) Nucleation overpotentials of Zn deposition on the PANZ@Cu and bare Cu in asymmetric cells (vs Zn electrode). B) CE of Zn plating/stripping in the Zn–Cu and PANZ@Zn‐PANZ@Cu half‐cells. C) Typical GCD profiles of Zn–Cu and PANZ@Zn‐PANZ@Cu half‐cells; morphology and zinc plating/stripping efficiency on a copper current collector; current density: 1 mA cm^−2^. D) Digital picture of current copper collector after plating for 2 h. E,F) SEM images of bare copper deposited zinc for 1 and 2 h, respectively. G) Magnified SEM image of zinc dendrite. H) Digital picture of copper current collector after plating for 2 h. I) SEM image of PAN@Cu after depositing Zn for 2 h. J) Peeled‐off PAN membrane. K) Cross‐section view SEM image of PAN@Cu after depositing Zn.

We also coated a PANZ coating layer on copper foil (PANZ@Cu) and tested two half cells (bare Zn||bare Cu and PANZ@Zn||PANZ@Cu) to investigate the effect of the PAN coating layer on the Coulombic efficiency (CE) of the zinc stripping/plating process and on the morphology of the deposited zinc (**Figure** **3**). The test was carried out by plating zinc (fixed areal capacity: 1 mAh cm^−2^) onto the Cu or PANZ@Cu substrate and then stripping to 1 V. The nucleation overpotential of PANZ@Cu (36 mV, Figure 3A) is much lower than that of bare Cu (51 mV), which is due to the considerably increased number of active nucleation sites on PANZ@Cu. As displayed in Figure 3B,C, the Zn||PANZ@Cu cell presents a very stable coulombic efficiency (CE) over 770 cycles (1440 h), with the average CE as high as 99.8% after the initial activation cycles. However, the voltage of the Zn||Cu cell showed sharp fluctuations after only 90 cycles (180 h) because of the internal short circuit in the cell caused by Zn dendrite formation.

To further reveal the effect of the PANZ coating layer on the deposition of zinc ions on the metal substrate, we investigated the morphology changes of the Zn deposited on Cu and PANZ@Cu, respectively. A phenomenon similar to that occurring on zinc foil was observed: the zinc was not uniformly deposited on the copper foil, and glass fibers adhered to the copper foil (Figure 3D). Many large‐sized nuclei and individual dendrites were observed after 1 h (Figure 3E), and the size and quantity of dendrites significantly increased after 2 h deposition (Figure 3F,G). However, the deposition on PANZ@Cu is very uniform, and the PANZ coating remains intact (Figure 3H,I). After uncovering the PANZ coating, a flat and dendrite‐free surface was observed (Figure 3J; and Figure S6A, Supporting Information), confirming that the PANZ coating does regulate the deposition of zinc metal. The cross‐section images of original PANZ@Cu (Figure S6B, Supporting Information) and zinc deposited PANZ@Cu (Figure 3K) show that the zinc deposition layer is uniform and dense, and the PANZ coating film remains intact. The EDS mapping further confirms the uniform deposition of zinc on PANZ@Cu (Figure S6C, Supporting Information). Furthermore, the X‐ray diffraction (XRD) results also show that the zinc deposit between the PANZ layer and Cu foil is a dense layer (Figure S7, Supporting Information).

**Figure 4:**
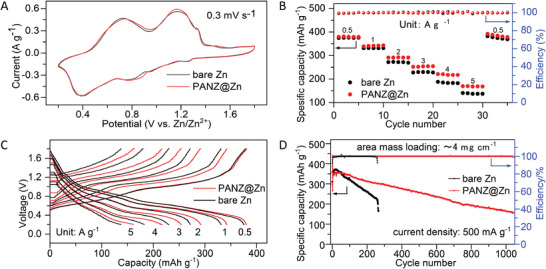
Electrochemical performance of full batteries with bare Zn||MnVO and PANZ @Zn||MnVO. A) Cyclic voltammetry of full batteries, scan rate 0.3 mV s^−1^. B) Rate performance of full batteries from 0.5 to 5 A g^−1^ based on the mass of MnVO. C) Capacity versus voltage curves at different current densities. D) Long‐term cycling performance of full batteries.

MnVO cathodes were investigated to further evaluate the impact of the PANZ coating layer on the electrochemical performance of full batteries consisting of bare Zn||MnVO and PANZ@Zn||MnVO cells (**Figure** **4**). MnVO was employed as cathode material to assemble a full battery due to the excellent reversibility, good electronic conductivity, and high specific capacity. The MnVO cathode material was synthesized by the hydrothermal method. The SEM image (Figure S8A, Supporting Information) and the XRD pattern are consistent with the reported MnVO literature, and show that this cathode can provide a proper interlayer distance for zinc ion intercalation/deintercalation.^[^ ^84^ ^]^

The CV curves of the two batteries were recorded between 0.2 and 1.8 V versus Zn/Zn^2+^ at a scan rate of 0.3 mV s^−1^. The two batteries, respectively, present two pairs of cathodic/anodic peaks located at 1.16/0.73 and 0.80/0.40 V (Figure 4A), corresponding to the zinc‐ion intercalation and deintercalation processes, respectively.^[^ ^84^, ^85^ ^]^ The CV curves of the MnVO cathode recorded at multiple scan rates (Figure S9, Supporting Information) show that the cathodic peaks are shifted toward more negative values with increasing scan rate, and the anodic peaks are shifted more positively, which can be ascribed to polarization.^[^ ^86^ ^]^

The rate performance of the Zn||MnVO and PANZ@Zn||MnVO batteries were also tested at various current densities ranging from 0.5 to 5 A g^−1^. As shown in Figure 4B, the rate performance of PANZ@Zn||MnVO is better than that of Zn||MnVO. The full battery PANZ@Zn||MnVO delivers a high specific capacity of 373 mAh g^−1^, 379, 340, 290, 254, and 220 mAh g^−1^ at current densities of 0.5, 1, 2, 3, and 4 A g^−1^, respectively. Even at a very high current density of 5 A g^−1^, the PANZ@Zn still displays a high discharge specific capacity of 170 mAh g^−1^, reaching ≈45% capacity utilization. However, the discharge specific capacity of the Zn||MnVO battery is lower than that of the PANZ@Zn||MnVO battery when the current density is over 1 A g^−1^. The charge–discharge curves in Figure 4C reveal that the PANZ@Zn||MnVO presents a smaller voltage hysteresis than Zn||MnVO, which can be attributed to the reduced interfacial impedance due to the improved wettability provided by introducing the PANZ coating layer.

The long‐term cycling stability of the full batteries was tested at 500 mA g^−1^ (Figure 4D). The battery with a bare Zn anode presented rapid capacity decay, and internal short circuit occurred at the 260th cycle (Figure S10A, Supporting Information). The fast decaying cycling performance can be attributed to the continuous corrosion of the unprotected Zn anode and the continuous growth of dendrites.^[^ ^87^ ^]^

By comparison, the cycling stability and lifespan of the PANZ@Zn||MnVO battery is enhanced significantly, with high capacity retention (255 mAh g^−1^) after 500 cycles and a longer lifespan of over 1000 cycles, even with the 20 µm thickness zinc foil used here. The dynamic EIS analysis of full batteries with PANZ@Zn||MnVO and Zn||MnVO (Figure S10B,C, Supporting Information) reveals that the battery with a PANZ@Zn anode has a slightly lower resistance than the Zn||MnVO battery, which can be attributed to the improvement of the wettability of Zn, thus reducing the interfacial impedance. The increase in impedance of Zn||MnVO with cycling confirms that the PANZ coating layer plays the key role of an artificial solid electrolyte interphase (SEI) to maintain impedance stability during battery cycling.

## 3 Conclusion

We have developed a PANZ coating layer consisting of PAN polymer and zinc salt on a Zn anode (PANZ@Zn) to suppress dendrite formation in ARZIBs. The zinc‐ion transport channels in the PANZ coating layer serve as a zinc‐ion flux regulator and enhance the uniform deposition of zinc ions on the zinc substrate. As a result, the PANZ coating layer reduces the interfacial impedance, resists dendrite formation, and alleviates side reactions on the zinc anode. Consequently, the PANZ@Zn anode in the symmetric batteries shows a dendrite‐free surface, and the symmetric cell has a longer lifespan than that of the uncoated bare zinc anode. Moreover, studies of the morphology and the reversibility on copper foil further affirm that the PANZ coating layer can promote the even electrodeposition of zinc ions and maintain high plating/stripping reversibility. The full battery with a PANZ@Zn anode has an excellent rate performance and a good cycling performance. The method proposed and the in‐depth understanding of the mechanism in the current work could represent a big step forward for zinc dendrite suppression and stimulate further efforts on zinc‐metal anodes and other aqueous metal‐ion batteries.

## Conflict of Interest

The authors declare no conflict of interest.

## Supporting information

Supporting Information

## Data Availability

The data that support the findings of this study are available from the corresponding author upon reasonable request.
