# Supplementary material for: An Artificial Polyacrylonitrile Coating Layer Confining Zinc Dendrite Growth for Highly Reversible Aqueous Zinc‐Based Batteries
Source: Adv Sci (Weinh). 2021 Mar 30;8(11):2100309. doi: 10.1002/advs.202100309 (PMC8188195; doi:10.1002/advs.202100309)
Supplement: Supplementary file 1 — Supporting Information [file ADVS-8-2100309-s001.pdf]

© 2021 Wiley-VCH GmbH

ADVANCED SCIENCE
Open Access

Supporting Information

for *Adv. Sci.,* DOI: 10.1002/advs.202100309

An Artificial Polyacrylonitrile Coating Layer Confining Zinc Dendrite Growth for Highly Reversible Aqueous Zinc-Based Batteries

Peng Chen [a, b], Xinhai Yuan [b], Yingbin Xia [b], Yi Zhang [b], Lijun Fu [, b], Lili Liu, Nengfei Yu [b], Qinghong Huang [b], Bin Wang [c], Xianwei Hu [*, a], Yuping Wu [*, b, c], Teunis van Ree [d]*

Supporting Information

An Artificial Polyacrylonitrile Coating Layer Confining Zinc Dendrite Growth for Highly Reversible Aqueous Zinc-Based Batteries

Peng Chen [a, b], *Xinhai Yuan* [b], *Yingbin Xia* [b], *Yi Zhang* [b], *Lijun Fu* [*, b], *Lili Liu*, *Nengfei Yu* [b], *Qinghong Huang* [b], *Bin Wang* [c], *Xianwei Hu* [*, a], *Yuping Wu* [*, b, c], *Teunis van Ree* [d]

[a] Key Laboratory for Ecological Metallurgy of Multimetallic Minerals (Ministry of Education), School of Metallurgy, Northeastern University, Shenyang 110819, China

[b] China State Key Laboratory of Materials-Oriented Chemical Engineering, School of Energy Science and Engineering, Nanjing Tech University, Nanjing 210009, China

[c] National Energy Novel Materials Center, Institute of Chemical Materials (ICM), China Academy of Engineering Physics (CAEP), No. 64, Mianshan Road, Mianyang 621900, China

[d] Department of Chemistry, University of Venda, Thohoyandou 0950, South Africa

*Corresponding authors.

E-mail: l.fu@njtech.edu.cn; huxw@smm.neu.edu.cn; wuyp@njtech.edu.cn

Experimental section

Preparation of electrodes. 1.0 g polyacrylonitrile (PAN, Shanghai RHWAN company, M.W.= 150,000) was added in 9.0 g dimethylformamide (DMF) while stirring (800 rpm) until the polymer completely dissolved, and then 1.0 g $Zn(TfO)_2$ was dissolved in the solution used to prepare a PANZ@Zn electrode. The same procedure was used to prepare a solution without zinc salt. Zinc foil with a thickness of 20 μm was purchased from the Qirui metal materials company in Hebei, and it was polished by sandpaper, and washed with distilled water and ethanol before use. The electrodes were coated with a PAN layer by putting 15 μL PAN solution (with and without $Zn(TfO)_2$) onto the zinc or copper disk (ϕ 10 mm). The as-prepared electrodes were dried on a hotplate at 55 °C to evaporate the solvent.

Synthesis of Mn-doped V_2O_5 (MnVO). 0.37 g V_2O_5 ordered from Sigma-Aldrich (> 99.6 %) was dissolved in 50 mL distilled water with 2 mL H_2O_2 (local vendor, 30%) added. 0.17 g $MnSO_4 \cdot H_2O$ (Sigma-Aldrich, ≥ 98%) was dissolved in 30 mL distilled water. The two solutions were then transferred to a 100 mL Teflon container in a stainless-steel autoclave that was heated to 120 °C at a rate of 5 °C min^{-1} and kept at 120 °C for 10 h. Brick-red precipitates were obtained by centrifugation and washed several times with distilled water followed by absolute ethanol. After drying in a vacuum oven at 80 °C for 12 h, the product's color changed to greenish.

Preparation of positive electrode. 30 mg colloidal dispersion containing 60 wt.% PTFE binder in distilled water was dispersed into 5 mL absolute ethanol by sonication. Then, 126 mg MnVO and 36 mg carbon black were transferred into the above prepared PTFE binder dispersion solution, which was then sonicated for 30 min to get a cathode slurry. The slurry was dried under an infrared lamp and then rolled into one piece, which was cut to 0.5 mm × 0.5 mm squares and then placed onto stainless-steel mesh. The area mass loading for the cathode electrode was ~4 mg cm^{-2}.

Contact angle measurements. The contact angle values of bare Zn, PAN coated Zn disk without any salt (PAN@Zn), and the PANZ@Zn electrode were measured on an optical contact angle goniometer (KINO INDUSTRY Co., Ltd., Model #SL200KS, USA). The contact angles ranging from 1° to 180° were tested with 4 μL of electrolyte at room temperature for 0 min, 4 min, 8 min, 12 min, 16 min, and 20 min, successively.

Ionic conductivity. Ionic conductivity of PANZ layer was measured by sandwiching the PANZ film wetted by 2 M $Zn(TfO)_2$ in two blocking electrodes (stainless steel sheets) for the electrochemical impedance spectroscopy (EIS) measurement with an amplitude of 10 mV over a frequency from 100kHz to 0.1 Hz (). The ionic conductivity was calculated by the following equation:

$$\sigma = \frac{100\, l}{RS} \qquad \text{(S-1)}$$

where *l* (mm) represents the thickness of PANZ layer and *S* (cm^2) is the area of the contact surface between one of the stainless sheets and the PANZ layer. *R* (Ω)is the intercept with real axis.

Material Characterization. The electrodes' morphology images were obtained using a desktop scanning electron microscope (SEM, Phenom). The X-ray diffraction (XRD) patterns of electrodes and MnVO were recorded using a Rigaku Smart Lab TM diffractometer using Cu Kα radiation.

Electrochemical measurement. All the electrochemical performance was investigated with CR2025 coin cells consisting of a working electrode, a counter electrode, and glass fiber (Whatman, grade GF/A, ≈260 μm) wetted with 2M $Zn(TfO)_2$ electrolyte (100 μL).

Galvanostatic charge/discharge cycling was performed on a Land (CT2001A Wuhan, China) battery-testing system.

Symmetric cells were assembled using zinc disks with a diameter of 10 mm and thickness of 20 μm in ambient atmosphere to evaluate the striping/plating performance of the PAN@Zn, PANZ@Zn, and bare zinc anodes. The long-term cycling performance and cycling stability of the zinc anodes were measured at a current density of 1mA cm^{-2} with fixed capacity of 1 mAh cm^{-2}.

The Coulombic efficiency (CE) of bare and coated zinc (bare Zn||bare Cu or PANZ@Zn||PANZ@Cu) was evaluated at 1 mA cm^{-2} and 1 mAh cm^{-2}. The cyclic voltammetry (CV) of the full batteries (bare Zn||MnVO or PANZ@Zn||MnVO) was measured using a CHI660D electrochemical workstation between 0.2 V and 1.8 V (vs Zn/Zn^{2+}) at 0.1 mVs^{-1}, 0.3 mVs^{-1}, 0.5 mVs^{-1}, 0.7 mVs^{-1}, 1 mVs^{-1} respectively. The rate performance of the full batteries (Zn||MnVO, PANZ@Zn||MnVO) was carried out at 0.5 A g^{-1}, 1 A g^{-1}, 2 A g^{-1}, 3 A g^{-1}, 4 A $g^{-1}$and 5 A g^{-1}. The current density and the specific capacity values of the electrodes were normalized to the mass of MnVO. Electrochemical impedance spectroscopy (EIS)

measurements were made on a CHI660D electrochemical workstation over the frequency range 100,000 Hz to 0.01 Hz with an AC voltage of 5 mV.

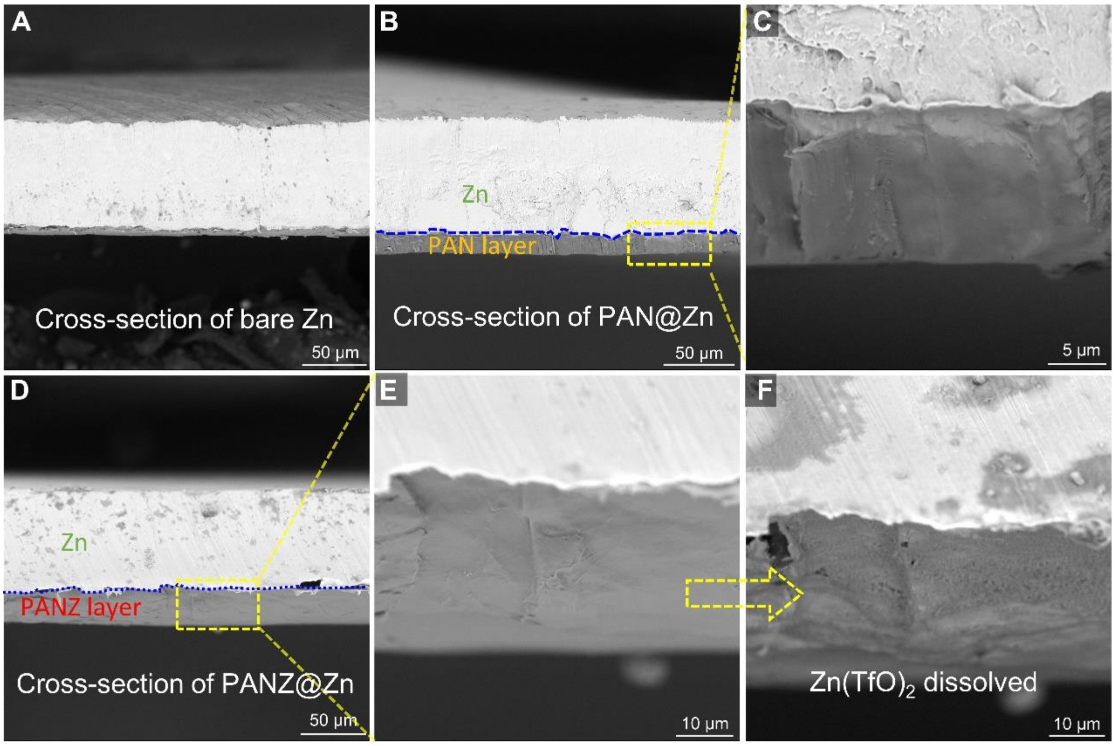

Figure S1 *Cross-section of bare zinc, PAN@Zn and PANZ@Zn anodes. (A) Bare Zn. (B-C) PAN@Zn. (D-E) PANZ@Zn. (F) The sample of (D) and (E) soaked in distilled water for 2 minutes and dried.*

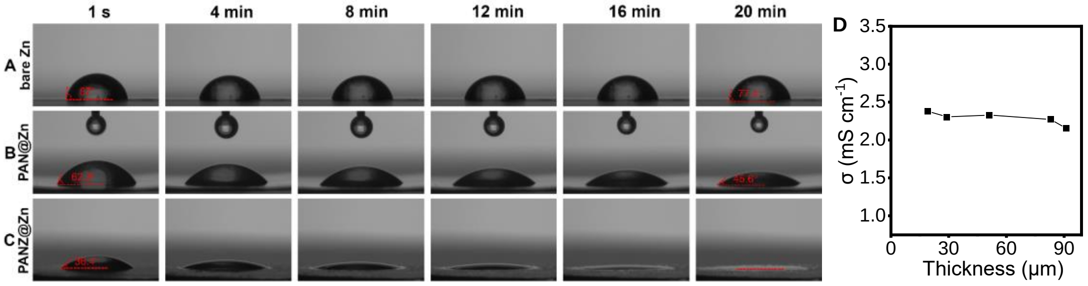

Figure S2 *Hydrophilicity and ionic conductivity. (A-C) Dynamic contact angles of bare Zn, PAN@Zn and PANZ@Zn. (D)Ionic conductivity of PANZ membranes with varying thickness.*

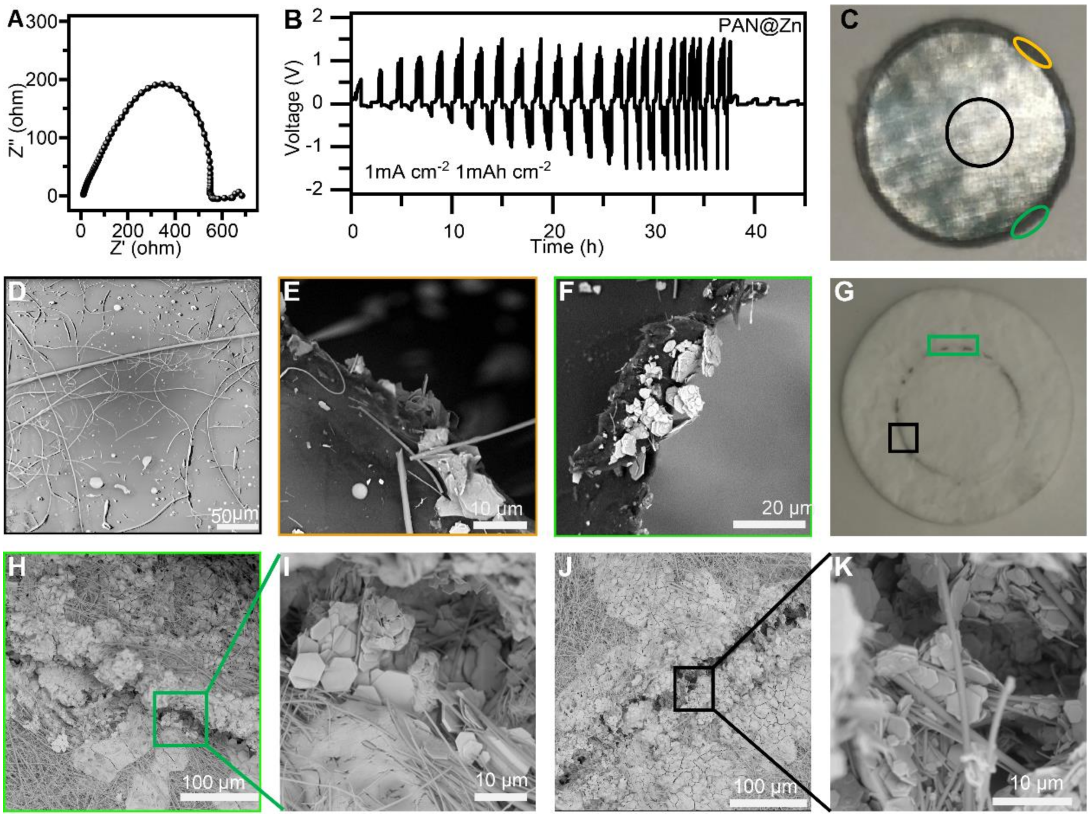

Figure S3 *Electrochemical performance and morphologies of PAN@Zn. (A) EIS of a symmetric cell with PAN@Zn||PAN@Zn. (B) Charge-discharge profile of a symmetric cell with PAN@Zn anode. (C) Digital picture of the PAN@Zn electrode disassembled from the failed symmetric cell. (D) SEM images of PAN@Zn (black dash-dotted line in (C). (E-F) SEM images of areas encircled by the brown and green lines in Figure S3C. (G) Digital picture of glass fiber separator disassembled from the same symmetric cell with electrode in B. (H-K) SEM images of separator pierced by dendrites.*

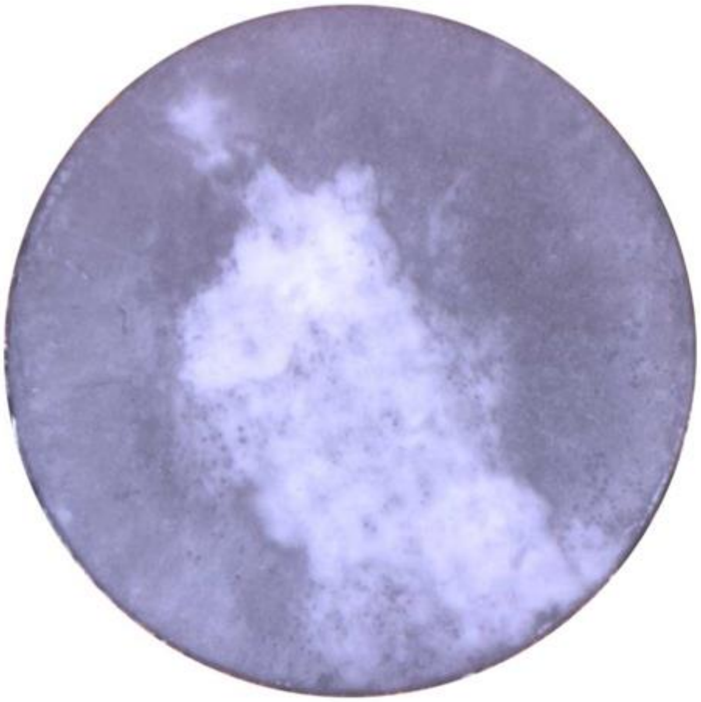

Figure S4 *Digital picture of Zn anode peeled off PANZ coating layer. The PANZ@Zn anode was cycled 100 cycles coupled with bare Zn. After the PANZ coating layer of the PANZ@Zn anode was peeled off, it was reassembled into coin cell and cycled for another 5 cycles at a current density of 1 mA cm^{-2} with a capacity of 1 mAh cm^{-2}.*

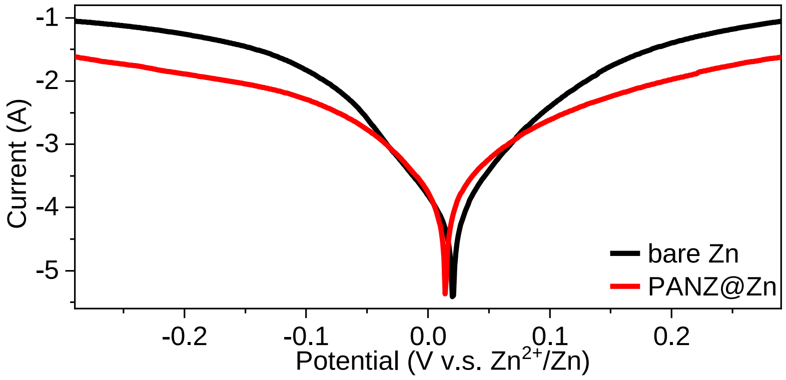

Figure S5 *Tafel curves of bare Zn and PAN@Zn in 2 M $Zn(TfO)_2$ electrolyte*

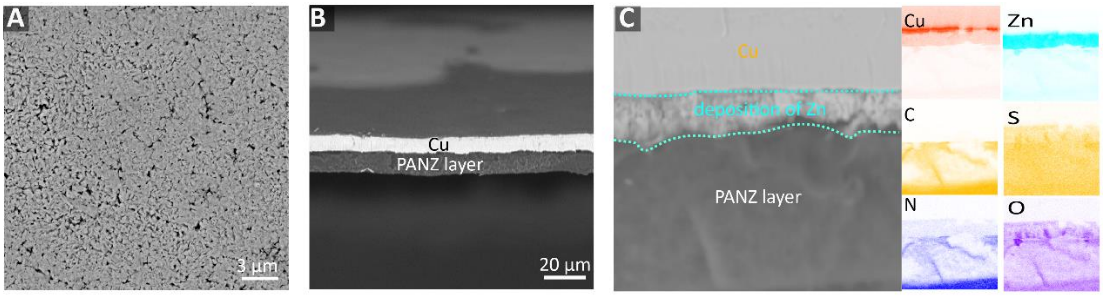

Figure S6 *Morphologies and elemental analysis of zinc deposition on copper foil. (A) Magnified SEM image of Zn deposition with a flat surface. (B) Cross-section view SME image of fresh PANZ@Cu, the thickness of PANZ coating layer is ≈ 11μm. (C) Cross-sectional SEM image of PANZ@Cu deposited Zn with EDS mapping of Cu, Zn, C, O, N, and S, respectively.*

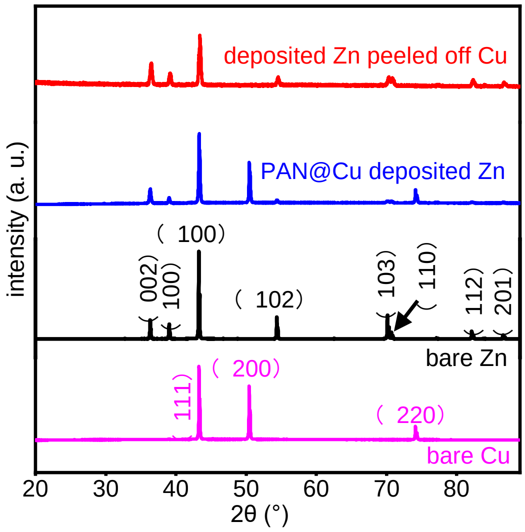

Figure S7 *XRD patterns of bare Cu, bare Zn, and PANZ@Cu deposited Zn, and deposition of zinc on PANZ@Cu without Cu.*

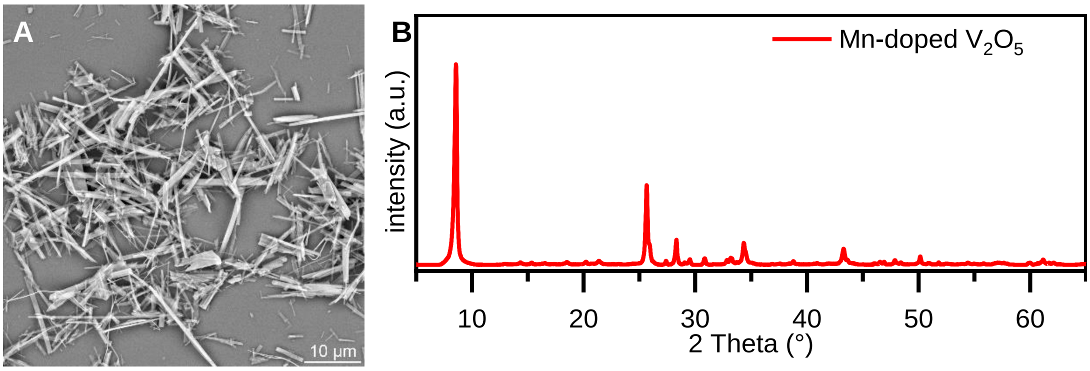

Figure S8 *(A) SEM image of prepared MnVO. (B) XRD pattern of MnVO.*

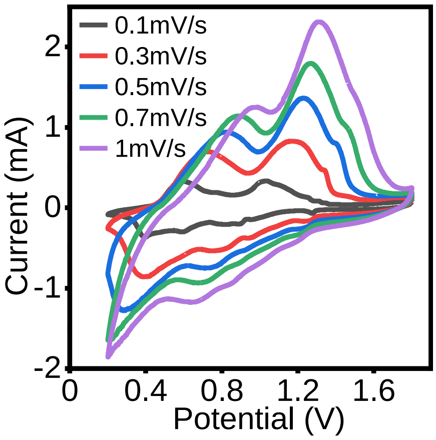

Figure S9 *CV curves of MnVO||PANZ@Zn at various scan rates*

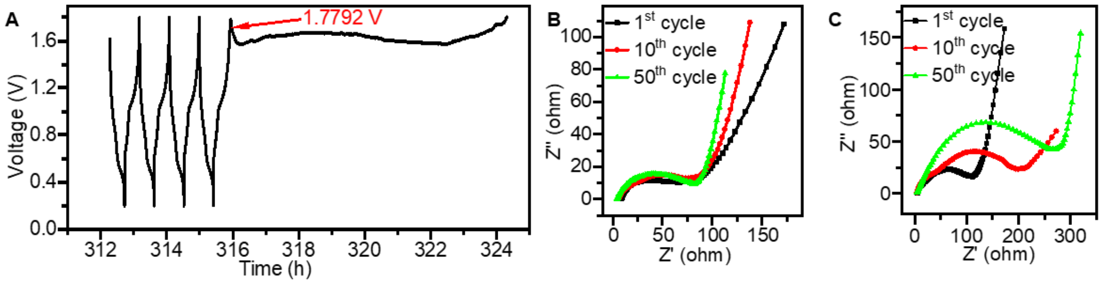

Figure S10 *Electrochemistry performance of full cells. (A) Charge-discharge curve of bare Zn || MnVO full battery which failed because of the internal short circuit. (B, C) electrochemistry impedance spectroscopy（EIS) of full batteries with PANZ@Zn and bare Zn anodes at 1*st*, 10*th *and 50*th *cycles, respectively.*

Table S 1 Different zinc anode modification methods and the corresponding electrochemistry performance

Zinc anodes	Current density (mA cm^{-2})	Areal capacity (mAh cm^{-2})	Worked time (h)	Reference (listed alphabetically)
nanoAu@Zn	0.25	0.05	2000	*ACS Appl. Energy Mater.* ***2019****, 2, 6490 – 6496*
rGO@Zn	1	2	200	*ACS Appl. Mater. Interfaces,* ***2018****, 10, 25446 - 25453*
C@Zn	1	1	200	*ACS Appl. Mater. Interfaces* ***2018****, 10, 22059 - 22066*
UIO-66@Zn	1	0.5	500	*ACS Appl. Mater. Interfaces,* ***2019****, 11, 32046 - 32051*
MoS_2@Zn	2.5	0.416	175	*ACS Appl. Mater. Interfaces* ***2020****, 12, 27249 - 27257*
nano$CaCO_3$@Zn	0.25	0.05	800	*Adv. Energy Mater.* ***2018****, 8, 1801090*
C@Zn (ZIF-8 pyrolysis)	1	1	400	*Adv. Energy Mater.* ***2020****, 10, 1904215*
Kaolin@Zn	4.4	1.1	800	*Adv. Funct. Mater.* ***2020,*** *30, 2000599*
PVB@Zn	0.5	0.5	2200	*Adv. Funct. Mater.* ***2020****, 30, 2001263*
ZrO_2@Zn	0.25	0.125	3700	*Adv. Funct. Mater.* ***2020****, 30, 1908528*
Pencil graphite@Zn	0.1	0.1	200	*Adv. Funct. Mater.* ***2021****, 31, 2006495*
ZnS@Zn	2	2	1100	*Adv. Mater.* ***2020****, 32, e2003021*
HsGDY@Zn	0.5	0.1	2400	*Adv. Mater.* ***2020****, 32, 2001755*
ZnF_2@Zn	1	1	800	*Adv. Mater.* ***2021****, 33, e2007388*
ALD-TiO_2@Zn	1	1	150	*Adv. Mater. Interfaces,* ***2018****, 5, 1800848*
HKUST-1@Zn	0.5	0.5	700	*Angew. Chem. Int. Ed.* ***2020****, 59, 2 - 7*
Nafion-Zn-X	1	0.5	1000	*Angew. Chem. Int. Ed.* ***2020****, 59,16594 - 16601*
ZIF-7@Zn	0.5	0.25	3000	*Angew. Chem. Int. Ed.* ***2020****, 59, 9377 - 9381*
In@Zn	0.25	0.05	1400	*Chem. Eng. J.* ***2020****, 396, 125363*
β-PVDF@Zn	0.25	0.05	2000	*Chem. Eng. J.* ***2021****, 411, 128584*
PAZ@Zn	0.5	0.25	8000	*Energy Environ. Sci.* ***2019****, 12, 1938 - 1949,*
ZnO@Zn	5	1.25	500	*Energy Environ. Sci.* ***2020****, 13, 503 - 510*
Cu@Zn	1	0.5	1500	*Energy Storage Mater.* ***2020****, 27, 205 - 211*
Sc_2O_3@Zn	0.5	0.5	275	*J. Energy Chem.* ***2021****, 55, 549 - 556*
ALD-Al_2O_3@Zn	1	1	500	*J. Mater. Chem. A* ***2020****, 8, 7836 - 7846*
PAM/PVP@Zn	0.2	0.1	2220	*J. Mater. Chem. A* ***2020****, 8, 17725 - 17731*
In situ ZnF_2-rich SEI@Zn	0.1	0.05	1000	*Nat. Commun.* ***2019****, 10, 5374*
Faceted TiO_2@Zn	1	1	460	*Nat. Commun.* ***2020****, 11, 3961*
In@Zn	1	1	500	*Small,* ***2020****, 16, e2001736*
PANZ@Zn	1	1	1145	**This work**
